# Activation of NF-E2 p45-related factor-2 transcription and inhibition of intestinal tumor development by AHCC, a standardized extract of cultured *Lentinula edodes* mycelia

**DOI:** 10.3164/jcbn.19-36

**Published:** 2019-09-27

**Authors:** Maiko Takahashi, Gen Fujii, Takahiro Hamoya, Yurie Kurokawa, Yui Matsuzawa, Kohei Miki, Masami Komiya, Takumi Narita, Michihiro Mutoh

**Affiliations:** 1 Division of Prevention, Center for Public Health Sciences, National Cancer Center, 5-1-1 Tsukiji, Chuo-ku, Tokyo 104-0045, Japan; 2 Central Radioisotope Division, National Cancer Center, 5-1-1 Tsukiji, Chuo-ku, Tokyo 104-0045, Japan; 3 Division of Carcinogenesis and Cancer Prevention, National Cancer Center, 5-1-1 Tsukiji, Chuo-ku, Tokyo 104-0045, Japan

**Keywords:** AHCC, oxidative stress, NRF2, IL-6, cancer chemoprevention

## Abstract

It has been reported that activation of NF-E2 p45-related factor-2 (NRF2), a transcription factor, induces a variety of antioxidant enzymes, and plays an important role in preventing carcinogenesis. AHCC is a standardized extract of cultured *Lentinula edodes* mycelia and it has been demonstrated to improve cancer. However, the effects of AHCC on NRF2 have not been examined, and the effects on intestinal adenoma development are not yet fully understood. We first investigated the effects of AHCC (1–5 mg/ml) on NRF2 activity in human colon cancer cell lines by a luciferase reporter gene assay, and found NRF2 transcriptional activities were increased ~12.6-fold. In addition, AHCC dose-dependently increased HO-1 and NQO-1 mRNA levels, and decreased interleukine-6 mRNA levels. Next, we administered 1,000 ppm AHCC for 8 weeks in the diet of *Apc* mutant Min mice, and found that AHCC significantly reduced the total number of intestinal polyps to 57.7% and to 67.6% of the control value in male and female Min mice, respectively, with suppression of interleukine-6 in the polyp part. These data suggest that AHCC possesses an ability to suppress cellular oxidative stress through activation of NRF2, thereby lowering intestinal polyp development in Min mice.

## Introduction

The estimated number of incident cases of colorectal cancer (CRC) shows it is the third most common cancer in men and second most in women worldwide in 2018.^(1)^ The trend of the estimated number of incident cases is decreasing, but the majority of CRCs occur in developed countries, including Japan.^(1)^ Thus, it is still important to establish useful methods for preventing CRC. One approach is using functional foods/plant-derived agents as cancer chemopreventive agents because many people can take them for long time through dietary habits.

Moreover, because of CRC’s natural history, it is effective to treat colorectal polyps at the stage of colorectal adenoma, a premalignant lesion, to prevent CRC development. It has been reported that interventions to detect and remove adenomas earlier are effective in reducing CRC cases and deaths.^(2,3)^ Thus, the target of functional foods/plant-derived agents could be colorectal adenoma.

It has been reported that oxidative stress is likely to play an important role in carcinogenesis through generation of reactive oxygen species (ROS), which modify DNA bases, induce DNA damage and lipid peroxidation.^(4)^ Under such oxidative stress, a variety of antioxidant enzymes are induced by activation of NF-E2 p45-related factor-2 (NRF2), a transcription factor.^(5,6)^ It is also reported that the proper basal function of NRF2 and Kelch-like ECH-associated protein 1 (KEAP1) is essential for preventing colorectal carcinogenesis.^(7)^ Nuclear factor-kappaB (NFκB) is another transcriptional factor induced by oxidative stress, and promotes carcinogenesis.^(8)^ Thus, reducing oxidative stress might be one of the targets of functional foods/plant-derived agents.

Previous studies have demonstrated that AHCC improve hyperlipidemia, obesity and cancer.^(9)^ AHCC is a standardized extract of cultured *Lentinula edodes* mycelia. This natural product is composed of a mixture of amino acids, minerals, polysaccharides and lipids enriched in α-1,4-linked glucan.^(9–11)^ It has been shown that AHCC stimulates immune response^(12–14)^ and has improved the prognosis and quality of life of several cancer patients.^(15–17)^ However, the effects of AHCC on NRF2 have not been examined, and the preventive action, i.e., the effect on intestinal adenoma development, is not yet fully understood.

In this study, we examined the antioxidant potential of AHCC by measuring oxidative stress-related transcription factors NRF2 and NFκB. We also investigated the effect of AHCC on the development of intestinal polyps in *Apc*-mutant Min mice, an animal model of human familial adenomatous polyposis that develops numerous adenomas in the intestinal tract.

## Materials and Methods

### Chemicals

AHCC^®^ is a standardized extract of cultured *Lentinula edodes* myceliais and is a trademark of Amino Up Co., Ltd. (Sapporo, Japan). AHCC^®^ is a gifted from Amino Up Co., Ltd. 2,2'-Azobis (2-methylpropion amidine) dihydrochloride (AAPH) is purchased from Tokyo Chemical Industry (Tokyo, Japan).

### Cell culture

HCT116 and DLD-1 cells, human colon adenocarcinoma cells, were purchased from the American Type Culture Collection (Manassas, VA). Both cell lines were maintained in DMEM supplemented with 10% heat-inactivated fetal bovine serum (FBS; HyClone Laboratories Inc., Logan, UT) and antibiotics (100 µg/ml streptomycin and 100 U/ml penicillin) at 37°C with 5% CO_2_.

### MTT assay

3-(4,5-Dimethylthiazol-2-yl)-2,5-diphenyltetrazolium bromide (MTT) was used to evaluate cell viability. HCT116 and DLD-1 colon cancer cells were seeded in 96-well plates (2 × 10^4^ cells/well). After 24 h incubation, the cells were treated with AHCC for 24 h. After 24 h, the cell medium was removed from the wells and 100 µl of 0.2 mg/ml MTT (Sigma, St. Louis, MO) was added to each well and incubated for 1 h at 37°C in 5% CO_2_. The supernatant was removed and crystals of formazan were dissolved by 100 µl/well DMSO. Thereafter, the absorbance at a wavelength of 595 nm was measured using a microplate reader.

### Luciferase reporter gene assay

HCT116 and DLD-1 colon cancer cells were transfected with NRF2-Luc or NFκB-Luc (Promega, Madison, WI) reporter plasmids using Polyethylenimine MAX MW 40,000 (PolySciences, Warrington, PA). The transfected cells were cultured for an additional 24 h. Cells stably expressing NRF2-Luc or NFκB-Luc were treated with hygromycin and cloned. These cells were referred to as HCT116-NRF2-Luc and DLD-1-NRF2-Luc cells, and HCT116-NFκB-Luc and DLD-1-NFκB-Luc respectively. HCT116-NRF2-Luc cells and DLD-1-NRF2-Luc cells were separately seeded in 96-well plates (2 × 10^4^ cells/well). After 24 h incubation, the cells were treated with AHCC for 24 h. Luciferase activity levels were determined using the Bright GLO Luciferase Assay System (Promega). In these cells, basal NRF2 and NFκB luciferase activities were normalized by protein assay. NRF2 and NFκB luciferase activity in the control was set as 1.0. Data are expressed as the mean ± SD (*n* = 4).

### Quantitative real-time polymerase chain reaction analyses

HCT116 and DLD-1 cells were seeded in 12-well plates (1.5 × 10^5^ cells/well). The cells were cultured in the presence of 0.5, 1, 2.5 and 5 mg/ml AHCC for 24 h. After 24 h, the mRNA expression levels regarding oxidative stress-related factors were evaluated by quantitative real-time polymerase chain reaction (PCR). Total RNA was isolated using RNAiso Plus (TaKaRa, Shiga, Japan). 100 ng aliquots in a final volume of 20 µl were used for synthesis of cDNA using a High Capacity cDNA Reverse Transcription Kit (Applied Biosystems, Foster City, CA) and oligo (dT) primers. Real-time PCR was carried out using the CFX96/384 PCR Detection System (BIO RAD, Tokyo, Japan) and Fast Start Universal SYBR Green Mix (Roche Diagnostics, Mannheim, Germany), according to the manufacturers’ instructions. The following primers were used to evaluate human mRNA levels: human IL-6 (5'-CTC GAG GAT GTA CCG AAT TTG T and 5'-ACT CAC CTC TTC AGA ACG AAT TG), human heme oxygenase-1 (HO-1) (5'-CCA GGC AGA GAA TGC TGA GT and 5'-GTA GAG AGG GGC GAA GAC TG), human NADP(H):quinone oxidoreductase-1 (NQO-1) (5'-CTG ATC GTA CTG GCT CAC TC and 5'-AAC AGA CTC GGC AGG ATA C) and human glyceraldehyde-3-phosphate dehydrogenase (GAPDH) (5'-TGC ACC ACC AAC TGC TTA GC and 5'-GGC ATG GAC TGT GGT CAT GAG). In addition, to assess the effects of AHCC on mRNA expression levels of inflammation-related factor in Min mice, we employed the primer sequences as follows: IL-6 (5'-ACA ACC ACG GCC TTC CCT ACTT and 5'-CAC GAT TTC CCA GAG AAC ATG TG), monocyte chemoattractant protein-1 (MCP-1) (5'-CCA CTC ACC TGC TGC TAC TCAT and 5'-TGG TGA TCC TCT TGT AGC TCT CC), and glyceraldehyde-3-phosphate dehydrogenase (GAPDH) (5'-TTG TCT CCT GCG ACT TCA and 5'-CAC CAC CCT GTT GCT GTA). A total of 12 µl in each well included 6 µl of 2× SYBR Green Master Mix, 6.4 µl of distilled water, 2 µl of the DNA sample (10 ng/µl) and 4 µl of primer set (1 µM each). Plates were run at 95°C for 10 min, and then 40 cycles of 95°C for 15 min and 60°C for 1 min. To assess the specificity of each primer set, the melting curves of the amplicons generated by the PCR reactions were analyzed.

### ROS assay

Cellular ROS levels were measured using the cell permeable probe 5-(and-6)-chloromethyl-2',7'-dichlorodihydrofluorescein diacetate (CM-H2DCFDA, Thermo Fisher, Waltham, MA). HCT116 and DLD-1 colon cancer cells were seeded in 96-well plates (2 × 10^4^ cells/well). After 24 h incubation, the cells were treated with AHCC for 24 h. After 24 h incubation, the cells were treated with AAPH for 3 h. After 3 h, the cell medium was removed from the wells and cells were loaded with 10 µM H2DCFDA in DMEM (phenol red-free) for 1 h. After washing the cells twice with DMEM, fluorescence was measured with a Synergy H1 Hybrid Multi-Mode Reader (BioTek, Winooski, VT).

### Animals

Male and female C57BL/6-*Apc*^Min/+^ mice (Min mice) were purchased from Jackson Laboratory (Bar Harbor, ME). The mice (*n* = 3–4) were housed in plastic cages with sterilized softwood chips as bedding in a barrier-sustained animal room maintained at 24 ± 2°C and 55% humidity under a 12 h light/dark cycle. The mice were fed an AIN-76A powdered basal control diet mixed with active hexose correlated compound at concentrations of 0, 250, 500, and 1,000 ppm.

Seven male Min mice and seven female Min mice aged 5 weeks were given 0 to 1,000 ppm AHCC for 8 weeks. All animals housed in the same cage were included in the same treatment group. Food and water were available *ad libitum*. The animals were observed daily for clinical symptoms and mortality. Body weight and food consumption were measured weekly. At the sacrifice time point, the mice were anesthetized, and blood samples were collected from their abdominal veins. Their intestinal tracts were removed and separated into the small intestine, cecum and colorectum. The small intestine was divided into a proximal segment (4 cm in length) and the rest of the segment containing proximal (middle) and distal halves. The number of polyps in the proximal segments were counted and collected under a stereoscopic microscope. The remaining intestinal mucosa (non-polyp part) was removed by scraping, and the specimens were stored at −80°C until quantitative real-time PCR analysis. The other regions were opened longitudinally and fixed flat between sheets of filter paper in 10% buffered formalin. Polyp numbers, size and intestinal distributions were assessed with a stereoscopic microscope. All experiments were performed according to the “Guidelines for Animal Experiments in the National Cancer Center” and were approved by the Institutional Ethics Review Committee for Animal Experimentation of the National Cancer Center. The animal protocol was designed to minimize pain and discomfort to the animals. The animals were acclimatized to laboratory conditions for more than two weeks prior to experimentation. All animals were euthanized by isoflurane overdose for tissue collection.

### Statistical analyses

Results are expressed as the mean ± SD, with statistical analyses performed using Dunnett’s test. Differences were considered to be statistically significant at *p<0.05, **p<0.01.

## Results

### Activation of NRF2 transcriptional activity by AHCC

**Fig. 1:**
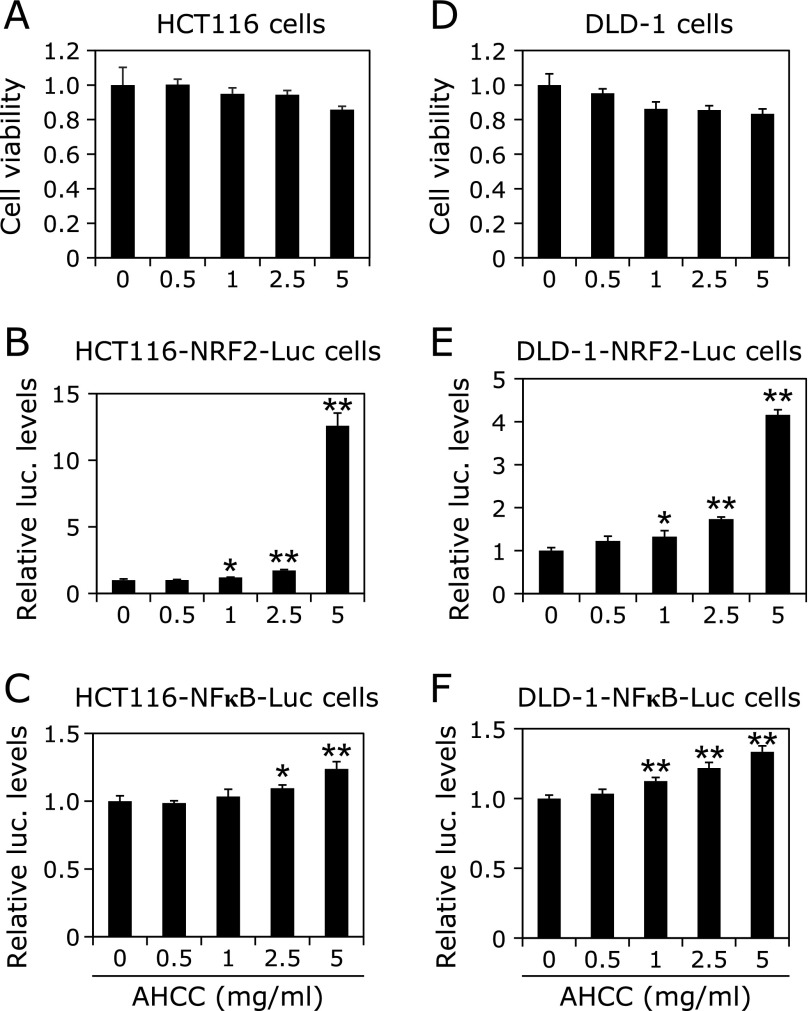
Effect of AHCC on NRF2 and NFκB promoter transcriptional activity in HCT116 and DLD-1 cells. HCT116 cells (A) and DLD-1 cells (D) treated with 0.5, 1, 5 and 10 mg/ml AHCC for 24 h were adopted for MTT assay. The luciferase activities of HCT116-NRF2-Luc cells (B) and DLD-1-NRF2-Luc cells (E) were measured after treatment with the indicated dose of AHCC for 24 h. The control basal luciferase activity level corrected by protein assay was set as 1.0. The luciferase activities of HCT116-NFκB-Luc cells (C) and DLD-1-NFκB-Luc cells (F) are also shown. Data are the mean ± SD (*n* = 3). *p<0.05, **p<0.01 vs control.

HCT116 and DLD-1 cells were treated with AHCC for 24 h (Fig. 1), and the effect evaluated on NRF2 and NFκB promoter transcriptional activities. In HCT116 cells, AHCC slightly decreased cell viability in a dose-dependent manner (Fig. 1A). Twenty-four hours treatment with 1, 2.5 and 5 mg/ml AHCC increased NRF2 promoter transcriptional activity 1.2 (*p*<0.05), 1.7 (*p*<0.01) and 12.6 (*p*<0.01)-fold as compared with the untreated control value, respectively (Fig. 1B). Besides, 24 h of 2.5 and 5 mg/ml AHCC treatment slightly increased NFκB promoter transcriptional activity by 1.1 (*p*<0.05), and 1.2 (*p*<0.01)-fold as compared with the untreated control value, respectively (Fig. 1C). In other colorectal cancer cells, DLD-1 cells, AHCC treatment slightly decreased the cell viability (Fig. 1D). Similarly, AHCC treatment increased NRF2 and NFκB promoter transcriptional activity (Fig. 1E and F).

### Effects of AHCC on mRNA expression levels regarding oxidative stress- and inflammation-related factors in HCT116 and DLD-1 cells

**Fig. 2:**
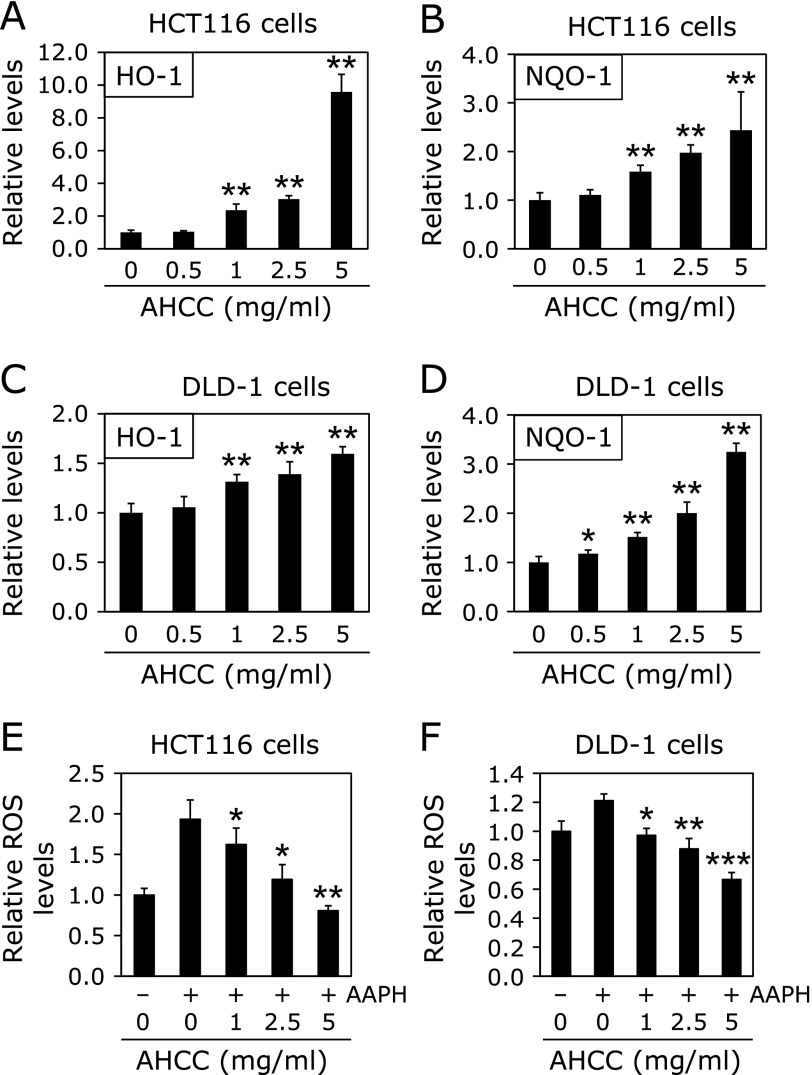
Expression levels of HO-1 and NQO-1 mRNA in HCT116 and DLD-1 cells treated with AHCC. HCT116 cells were seeded in 12-well plates (1.5 × 10^5^ cells/well) and cultured in medium containing 0.5, 1, 2.5 and 5 mg/ml AHCC for 24 h. After 24 h, mRNA expression levels of HO-1 (A) and NQO-1 (B) were evaluated by quantitative real-time PCR analysis. Change of HO-1 (C) and NQO-1 (D) mRNA expression levels were also shown in DLD-1 cells. The basal mRNA expression levels of the control were set as 1.0. Data were normalized with GAPDH. Data are the mean ± SD (*n* = 3). *p<0.05, **p<0.01 vs 0 control. HCT116 cells (E) and DLD-1 cells (F) were seeded in 96-well plates (2.0 × 10^4^ cells/well) and cultured in medium containing 10 mM AAPH for 3 h after treated with 1, 2.5 and 5 mg/ml AHCC for 24 h. After treated with AAPH, ROS levels were evaluated by DCFDA. Data are the mean ± SD (*n* = 4). *p<0.05, **p<0.01, ***p<0.001 vs 0 mg/ml AHCC with 10 mM AAPH.

In order to clarify the effects of AHCC on the downstream targets of NRF2, mRNA expression regulated by NRF2 was evaluated in HCT116 and DLD-1 cells. AHCC significantly increased HO-1 and NQO-1 mRNA levels in a dose-dependent manner in HCT116 cells (Fig. 2). Twenty-four hours of 1, 2.5 and 5 mg/ml AHCC treatment significantly increased 2.4 (*p*<0.01), 3.0 (*p*<0.01) and 9.6 (*p*<0.01)-fold of HO-1 mRNA levels compared with the untreated control value, respectively, and increased 1.6 (*p*<0.01), 2.0 (*p*<0.01) and 2.4 (*p*<0.01)-fold of NQO-1 mRNA levels, respectively (Fig. 2A and B). In DLD-1 cells, the increase of HO-1 mRNA levels by AHCC treatment was weaker than that of HCT116 cells (Fig. 2C). However, NQO-1 mRNA expression was similarly increased compared to those of HCT116 cells, 1.2 (*p*<0.05), 1.5 (*p*<0.01), 2.0 (*p*<0.01) and 3.3 (*p*<0.01)-fold (Fig. 2D). In addition, AAPH (a radical starter)-produced ROS was detected in the medium cultured HCT116 and DLD-1 cells. In this condition, 24 h of 1, 2.5, 5 mg/ml AHCC treatment significantly decreased AAPH-produced ROS levels (Fig. 2E and F). However, AHCC plus AAPH treatment did not clearly affect NRF2 and NFκB transcriptional activities (Supplemental Fig. 1*).

**Fig. 3:**
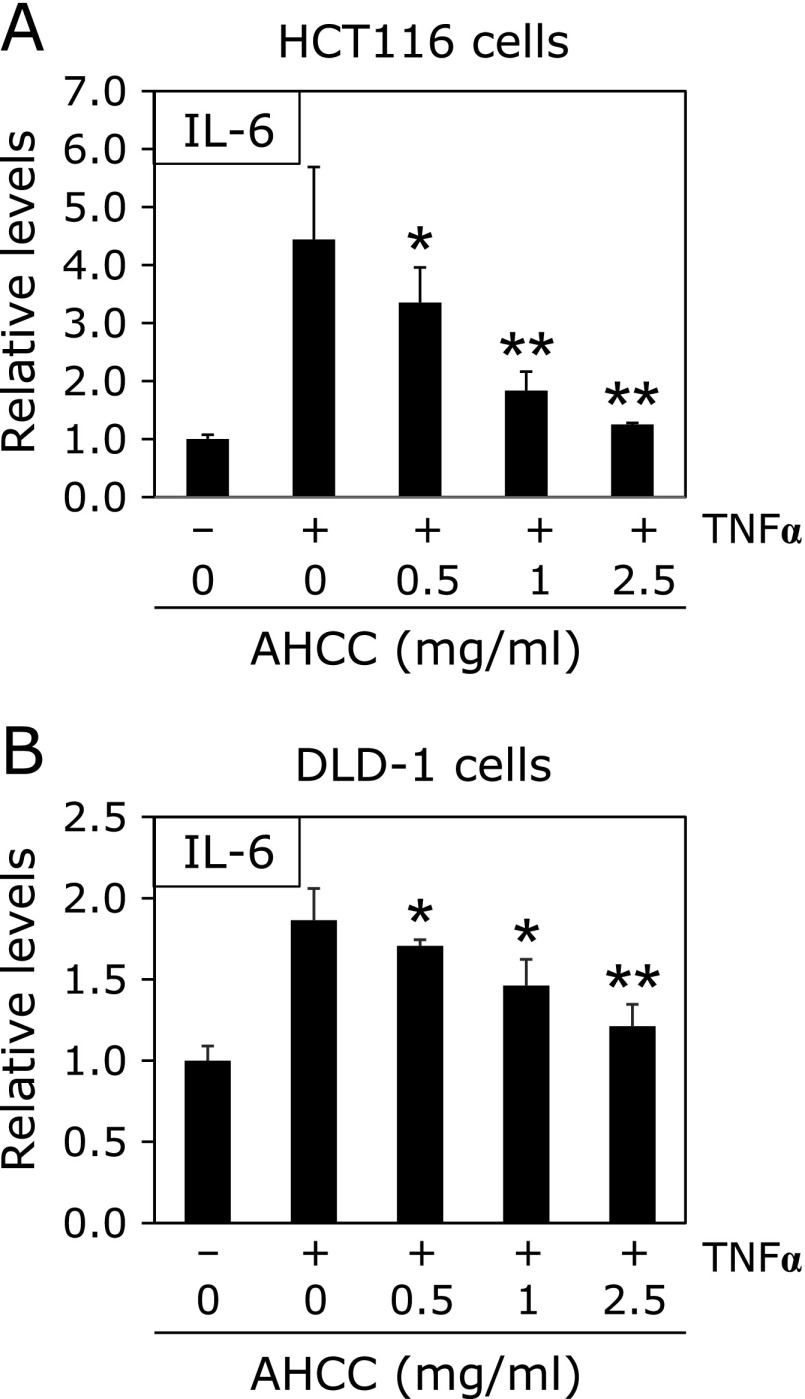
Effects of AHCC on IL-6 mRNA expression levels with TNFα in HCT116 and DLD-1 cells. HCT116 and DLD-1 cells were seeded in 12-well plates (1.5 × 10^5^ cells/well) and cultured in medium containing AHCC (0.5, 1, 2.5 and 5 mg/ml) with TNFα (10 ng/ml) for 24 h. After 24 h, IL-6 mRNA expression levels in HCT116 cells with TNFα (A), and in DLD-1 cells with TNFα (B) were evaluated by quantitative real-time PCR analysis. The basal mRNA expression levels of the control were set as 1.0. Data were normalized with GAPDH. Data are the mean ± SD (*n* = 3). *p<0.05, **p<0.01 vs 0 mg/ml AHCC with 10 ng/µl TNFα.

To clarify the effects of AHCC on the downstream targets of NRF2, IL-6 mRNA expression levels were evaluated in HCT116 and DLD-1 cells following 24 h of 0.5, 1, 2.5 mg/ml AHCC and 10 ng/µl TNFα treatment. In HCT116 cells, 24 h of AHCC treatment significantly decreased IL-6 mRNA expression levels under 10 ng/ml TNFα stimulation (Fig. 3A). Similar results were obtained in DLD-1 cells (Fig. 3B). AHCC treatment increased TNFα-stimulated IL-6 mRNA expression levels by 36% (*p*<0.01) compared with the untreated control value.

### Inhibition of intestinal polyp development in Min mice by AHCC

Administration of AHCC to Min mice for 8 weeks did not affect body weight, food intake or clinical symptoms throughout the experimental period. There was no difference in average daily food intake between the basal diet control group and AHCC-treated groups of Min mice. In addition, no changes in organ weights that may have been attributable to toxicity were observed. Table 1 summarizes the data regarding the numbers and distributions of intestinal polyps in the basal diet control group and AHCC-treated groups.

The number of polyps was mostly observed in the small intestine, and some did not have polyps in the colon. Treatment with 1,000 ppm AHCC significantly reduced the total number of intestinal polyps to 57.7% of the control value in male Min mice and to 67.6% of the control value in female Min mice (*p*<0.01 vs control group).

In treatment with 500 ppm AHCC for males, a reduction in the number of polyps to 34.5% of the basal control diet group was observed in the proximal segment (*p*<0.01 vs control group), and a reduction in the number of polyps to 57.6% of the basal control diet group was observed in the middle segment of the small intestine (*p*<0.01 vs control group). In administration of 1,000 ppm AHCC for males, a reduction in the number of polyps to 54.8% of the basal control diet group was observed in the proximal segment (*p*<0.05 vs control group), and a reduction in the number of polyps to 60.0% of the basal control diet group was observed in the middle segment of the small intestine (*p*<0.01 vs control group). In administration of 500 ppm AHCC for females, a reduction in the number of polyps to 60.2% of the basal control diet group was observed in the proximal segment of the small intestine (*p*<0.05 vs control group). In administration of 1,000 ppm AHCC for females, a reduction in the number of polyps to 62.7% of the basal control diet group was observed in the proximal segment of the small intestine (*p*<0.05 vs control group). For male Min mice, a remarkable decrease in number of polyps was observed in the all segments of the small intestine (*p*<0.05 vs control group), whereas in female Min mice, reduction was only observed in the distal segment of the small intestine.

Supplemental Fig. 2* shows the size distributions of the intestinal polyps in the basal diet and AHCC-treated groups. AHCC treatment for male Min mice significantly reduced the numbers of polyps <1.5 mm in diameter (*p*<0.05 vs control group). Similarly, AHCC treatment for female Min mice significantly reduced the numbers of polyps <1.0 mm in diameter (*p*<0.05 vs control group).

**Fig. 4:**
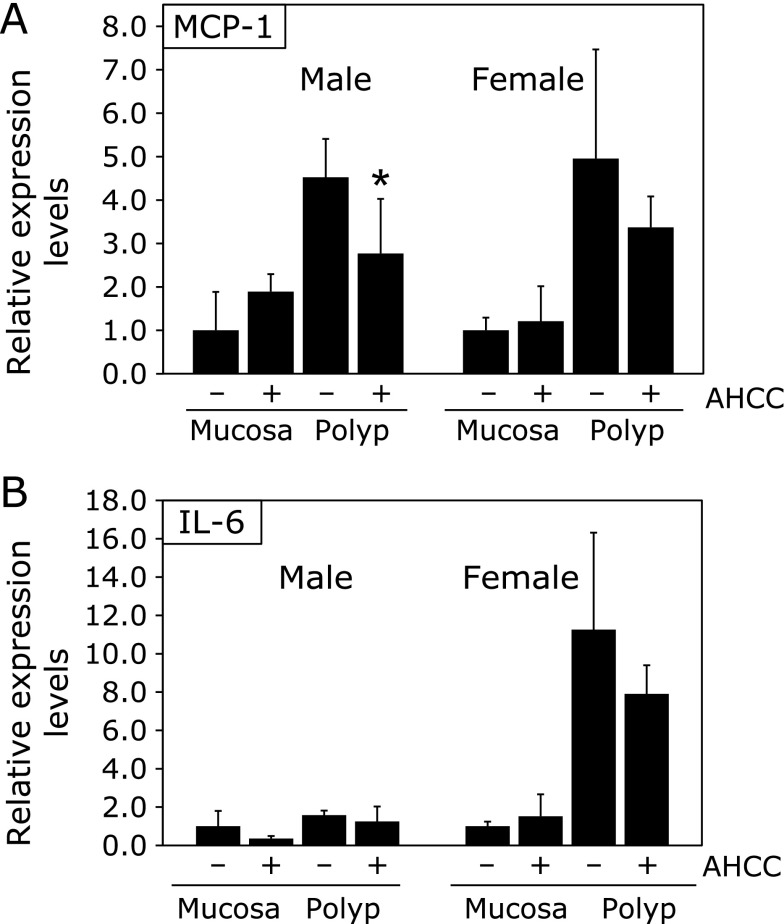
Inflammation-related mRNA expression levels in the intestines of Min mice. Quantitative real-time PCR analysis was performed to determine MCP-1 mRNA (A) and IL-6 mRNA (B) expression levels in the non-polyp (mucosa) and polyp part of the intestines of Min mice. Data were normalized with GAPDH. Each expression level in the non-polyp part of the intestines in the control group (0 ppm) was set as 1. Data are the mean ± SD (*n* = 4). *p<0.05 vs control group.

To assess the mechanisms underlying AHCC-mediated suppression of intestinal polyp formation, we evaluated the downstream targets of NRF2, such as MCP-1, and IL-6 mRNA expression in the non-polyp (mucosa) and polyp part of the intestine was investigated (Fig. 4). Real-time PCR revealed that treatment with 1,000 ppm AHCC for 8 weeks suppressed MCP-1 mRNA expression levels in the intestinal polyp of male and female mice by 39% (*p*<0.05), and 32% respectively, compared with the untreated control values (Fig. 4A). Furthermore, AHCC treatments tended to decrease IL-6 mRNA expression levels in the intestinal polyp part of males and females (Fig. 4B). In the non-polyp (mucosa) portion, no difference of MCP-1 and IL-6 mRNA expression between the AHCC treatment and non-treatment group was observed. However, it was interesting to observe that there was tendency to elevated MCP-1 and IL-6 mRNA expression in the mucosa.

## Discussion

In the present study, AHCC was shown to significantly activate one of the oxidative stress-related transcriptional factors, NRF2, in a human colon cancer cell line. Moreover, AHCC prevented intestinal polyp development formation in *Apc*-mutant Min mice with reduced IL-6 expression levels in the polyp part.

AHCC has been shown to inhibit constitutive signal transducer and activator of transcription 3 (STAT3) phosphorylation in ovarian cancer cells.^(18)^ However, AHCC has not been shown to increase NRF2 transcriptional activity hitherto. Thus, this is the first report that shows significant elevation of NRF2 transcriptional activity by AHCC treatment *in vitro*. The significance of increased NRF2 transcriptional activity is further highlighted by previous studies demonstrating that NRF2 is an important molecular target for naturally derived cancer chemopreventive agents both *in vitro* and *in vivo*.^(19)^ It has been reported that 5% tomato powder decreases the development of aberrant crypt foci (ACF) and adenocarcinoma, along with regulating NRF2/HO-1 signaling pathway in the colorectal tissue of azoxymethane (AOM)-treated Wistar rats.^(19)^ Our previous reports also demonstrated that citrus peel extract significantly activated NRF2, in a human colon cancer cell line, and prevented AOM-induced ACF formation in the colon of F344 rats via antioxidant action.^(20)^

Our present data also demonstrate that AHCC increases the transcriptional activity of NF-κB weakly, but significantly. Inconsistent with our data, other reports demonstrated that AHCC did not influence the activation of NF-κB stimulated by IL-1β.^(21)^ We do not know the reason exactly but assumed that weak suppression of cell viability induced by AHCC may secondarily activate NF-κB transcriptional activity since an increase in NF-κB transcriptional activity confers an antiapoptotic phenotype through inhibition of apoptosis protein (IAP) family induction.^(22)^

We confirmed that our increase of NRF2 transcriptional activity is functionally working by detecting downstream mRNA expression levels. Actually, AHCC treatment significantly decreased AAPH-produced ROS levels. NRF2 is a helix-loop-helix basic leucine zipper transcription factor that binds to the antioxidant response element (ARE), which results in transcriptional activation of genes involved in carcinogen detoxication and attenuation of oxidative stress, such as glutathione *S*-transferase (GST), UDP-glucuronosyltransferase (UGT), HO-1, NQO and gamma glutamylcysteine synthetase (γGCS).^(23)^ We showed in the present study that AHCC significantly increased HO-1 and NQO-1 mRNA levels in a dose-dependent manner in human colon cancer cells.

The reason why we used Min mice is that these mice are known to be under high oxidative stress. We demonstrated that oxidant-related triglycerides and oxidized phosphatidylcholines, such as aldehyde and carboxylic acid types, were increased in the serum of Min mice.^(24)^ Moreover, we showed that NADPH oxidase 1 (NOX1) and p22^phox^ mRNA levels tend to be elevated in the intestinal tumors of Min mice.^(25)^ NOX1 and p22^phox^ belong to a nicotinamide adenine dinucleotide phosphate (NADPH) oxidase family that produces ROS. Thus, it is assumed that this mouse is an appropriate model to observe the antioxidative effects of AHCC on tumorigenesis *in vivo*.

We did not uncover the mechanisms why AHCC inhibited intestinal polyp development. However, suppression of MCP-1 and IL-6 mRNA levels may affect the results. MCP-1 and IL-6 are the cytokines primarily produced at acute and chronic inflammation sites, and act as both pro-inflammatory and anti-inflammatory cytokines. The function of the MCP-1 and IL-6 gene is implicated in a wide variety of inflammation-associated diseases, such as cancer.^(26,27)^ In Min mice, lacking the IL-6 gene decreased overall polyp number by 32% compared to that of IL-6 wild Min mice. The Min mouse showed a 10-fold increase in serum IL-6 levels at 26 weeks of age.^(28)^ Thus, down regulation of IL-6 in polyps may be involved in the inhibition of intestinal polyp development. It has been reported that NRF2 regulates oxidative stress-response, and represses inflammation, including suppression of MCP-1 and IL-6.^(29,30)^ Thus, our speculation of an NRF2-MCP-1 and IL-6 axis in the suppression of polyp development is not something unusual. Further research is needed to confirm this possibility.

In conclusion, our data suggest that AHCC may block cellular oxidative stress through induction of NRF2, thereby preventing intestinal polyp development in Min mice. It was worthwhile to show first time in this study that AHCC has a preventive effect other than a therapeutic one. The use of AHCC as a functional food/plant-derived agent may favor prevention of colorectal cancer. Further mechanisms of preventing intestinal polyp development by AHCC will be revealed in future studies.

## Figures and Tables

**Table 1:** Numbers of intestinal polyps in the basal diet group and AHCC treated diet group in Min mice

Number of intestinal polyps/mouse in male Min mice						
AHCC (ppm)	No. of mice	Small intestine			Colon	Total
		Proximal	Middle	Distal		
0	7	8.4 ± 3.3	14.4 ± 4.1	34.7 ± 13.0	2.0 ± 2.4	58.7 ± 18.1
250	7	3.6 ± 1.6*	10.7 ± 4.8	26.7 ± 7.7	1.1 ± 0.9	42.1 ± 11.2
500	7	2.9 ± 1.6*	8.3 ± 2.1*	21.4 ± 6.1^#^	0.3 ± 0.5	32.9 ± 5.8*
1,000	7	4.6 ± 1.0^#^	8.6 ± 3.7*	21.6 ± 5.3^#^	0.9 ± 0.9	33.9 ± 8.2*

Data are mean ± SD. *Significantly different from the control untreated group at *p*<0.01. ^#^*p* = 0.05 vs 0 ppm.						

Number of intestinal polyps/mouse in female Min mice						
AHCC (ppm)	No. of mice	Small intestine			Colon	Total
		Proximal	Middle	Distal		
0	7	4.4 ± 1.4	12.4 ± 3.4	28.7 ± 10.9	0.6 ± 0.5	47.3 ± 11.0
250	6	3.3 ± 1.6	8.8 ± 3.1	25.5 ± 6.3	0.5 ± 0.5	38.7 ± 9.0
500	7	5.0 ± 2.0	11.9 ± 5.0	17.3 ± 6.8^#^	0.4 ± 0.8	34.6 ± 13.3
1,000	7	4.3 ± 2.0	9.1 ± 3.5	18.0 ± 4.8^#^	0.6 ± 0.5	32.0 ± 6.1*

Data are mean ± SD. *Significantly different from the control untreated group at *p*<0.01. ^#^*p* = 0.05 vs 0 ppm.						

## Abbreviations

ACF: aberrant crypt foci
AOM: azoxymethane
ARE: antioxidant response element
CRC: colorectal cancer
FBS: fetal bovine serum
γGCS: gamma glutamylcysteine synthetase
GAPDH: glyceraldehyde-3-phosphate dehydrogenase
GST: glutathione *S*-transferase
HO-1: heme oxygenase-1
IAP: inhibitor of apoptosis protein
IL: interleukin
KEAP1: Kelch-like ECH-associated protein 1
MCP-1: monocyte chemoattractant protein-1
MTT: 3-(4,5-dimethylthiazol-2-yl)-2,5-diphenyltetrazolium bromide
NADPH: nicotinamide adenine dinucleotide phosphate
NFκB: nuclear factor-kappaB
NQO: NADP(H):quinone oxidoreductase
NRF2: NF-E2 p45-related factor-2
PCR: polymerase chain reaction
ROS: reactive oxygen species
STAT3: signal transducer and activator of transcription 3
UGT: UDP-glucuronosyltransferase
