# Supplementary material for: Activation of NF-E2 p45-related factor-2 transcription and inhibition of intestinal tumor development by AHCC, a standardized extract of cultured Lentinula edodes mycelia
Source: J Clin Biochem Nutr. 2019 Sep 27;65(3):203–8. doi: 10.3164/jcbn.19-36 (PMC6877408; doi:10.3164/jcbn.19-36)
Supplement: Supplemental Figure 1 [file jcbn19-36sf01.pdf]

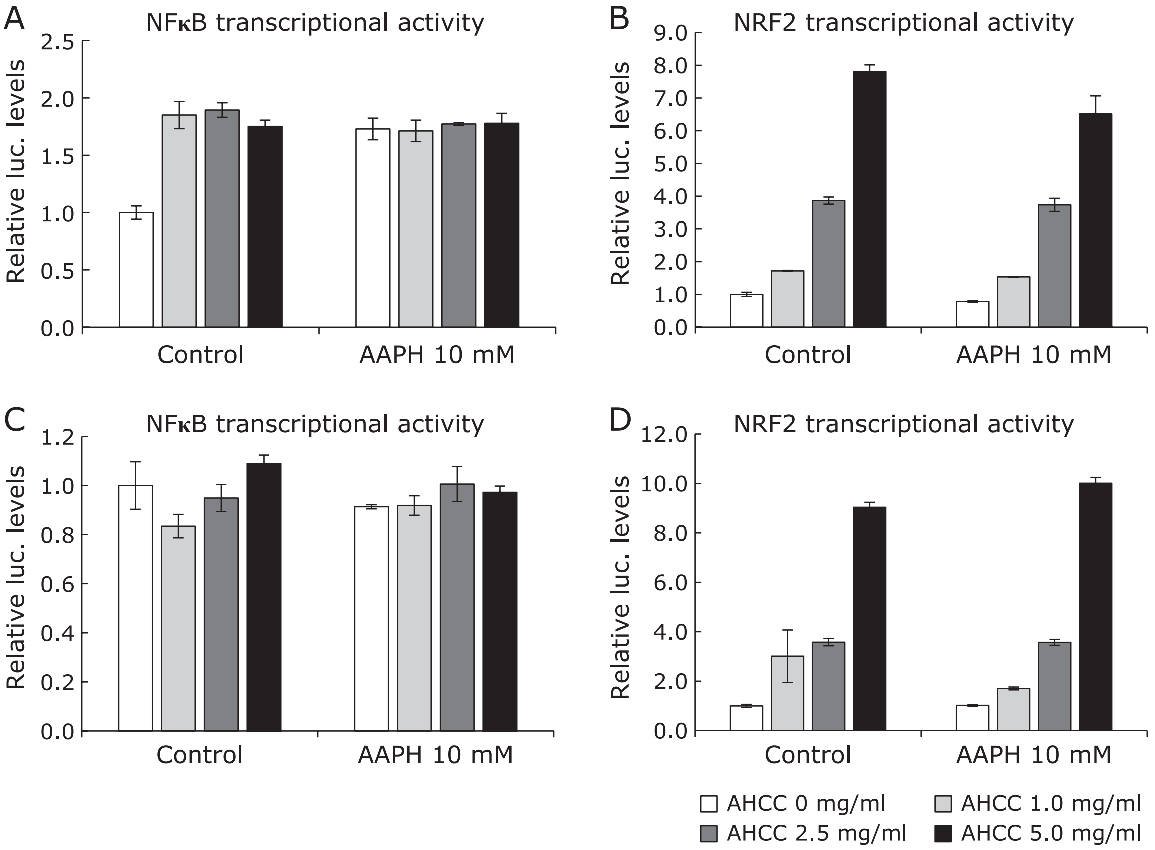

Supplemental Fig. 1. Effect of AHCC on NRF2 and NFκB promoter transcriptional activity in HCT116 and DLD-1 cells. HCT116 cells (A, B) and DLD-1 cells (C, D) were seeded in 96-well plates (2.0 × 104 cells/well) and cultured in medium containing 10 mM AAPH for 3 h after treated with 0.5, 1, 2.5 and 5 mg/ml AHCC for 24 h. After treated with AAPH, the luciferase activity was measured. The control basal luciferase activity level corrected by protein assay was set as 1.0.
