# Supplementary material for: Activation of NF-E2 p45-related factor-2 transcription and inhibition of intestinal tumor development by AHCC, a standardized extract of cultured Lentinula edodes mycelia
Source: J Clin Biochem Nutr. 2019 Sep 27;65(3):203–8. doi: 10.3164/jcbn.19-36 (PMC6877408; doi:10.3164/jcbn.19-36)
Supplement: Supplemental Figure 2 [file jcbn19-36sf02.pdf]

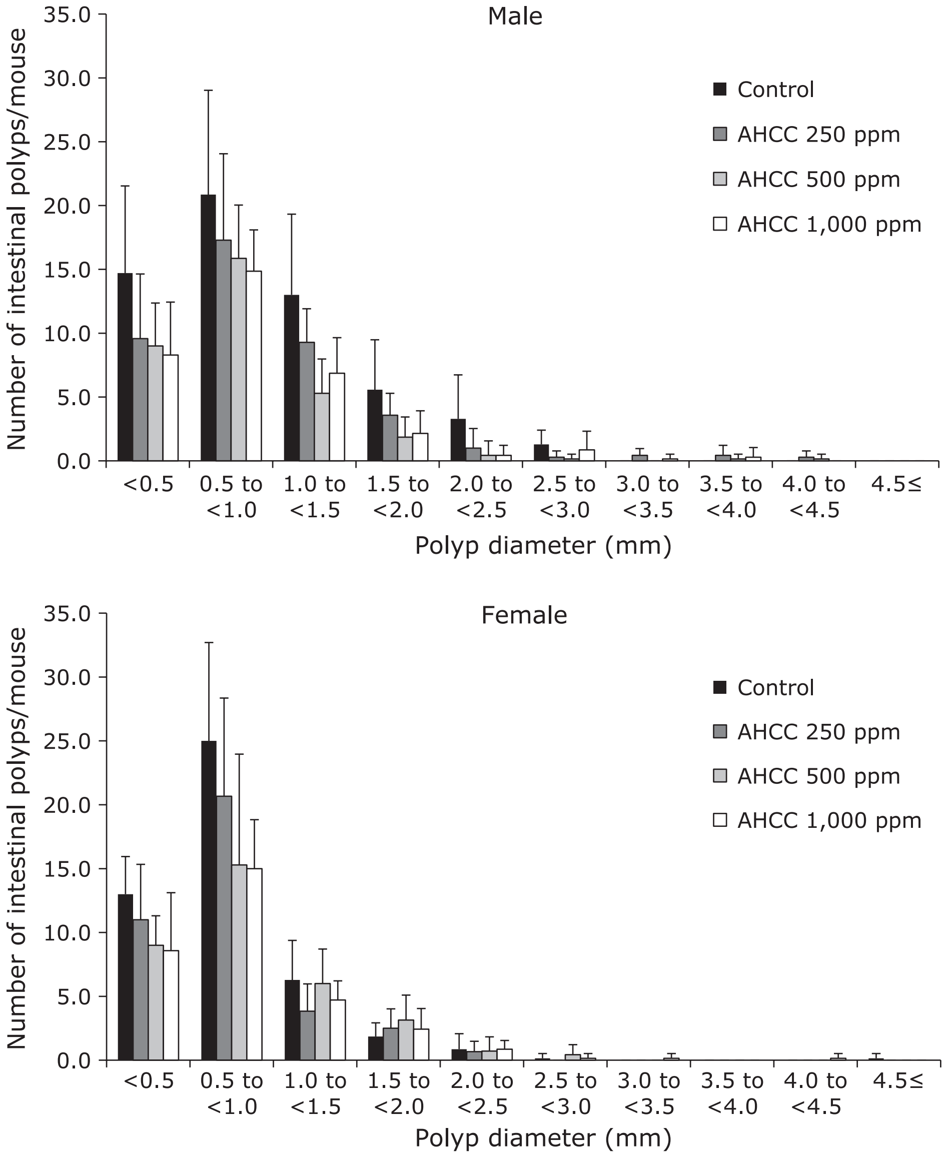

Supplemental Fig. 2. Male and female Min mice were fed a basal diet (open box) or a diet containing 250–1,000 ppm (filled box) AHCC for 8 weeks. The number of polyps per mouse in each size class is given as the mean ± SD.
